# Distal Gastrectomy With Roux-en-Y Reconstruction for a Seriously Dilated Stomach With Gastric Outlet Obstruction Secondary to Sarcina ventriculi: A Case Report

**DOI:** 10.7759/cureus.35523

**Published:** 2023-02-27

**Authors:** Hazem Abosheaishaa, Mahmoud Nassar, Bahaaeldin Baraka, Mostafa Alfishawy, Asad Sahibzada

**Affiliations:** 1 Internal Medicine, Icahn School of Medicine at Mount Sinai, Queens Hospital Center, New York, USA; 2 Internal Medicine/Gastroenterology, Cairo University, Cairo, EGY; 3 Internal Medicine, Icahn School of Medicine at Mount Sinai/NYC Health and Hospitals Queens, New York, USA; 4 Oncology, University Hospitals of Leicester NHS Trust, Leicester, GBR; 5 Infectious Diseases, Infectious Diseases Consultants and Academic Researchers of Egypt (IDCARE), Cairo, EGY; 6 Pulmonary and Critical Care, Icahn School of Medicine at Mount Sinai, Queens Hospital Center, New York, USA

**Keywords:** gastric outlet obstruction, gastroparesis, intestinal pseudo-obstruction, intestinal obstruction, sarcinia ventriculi

## Abstract

*Sarcina ventriculi* is an anaerobic gram-positive coccus that can resist the acidic media of the stomach and cause gastrointestinal symptoms. Here, we report the case of a 43-year-old male patient with a history of schizophrenia presenting with abdominal distention, nausea, vomiting, early satiety, and weight loss. Computed tomography of the abdomen and pelvis with contrast revealed a severely dilated stomach and signs of gastric outlet obstruction on multiple occasions. The endoscopic evaluation showed a dilated stomach, and biopsies revealed non-specific gastritis, negative *Helicobacter pylori*, and positive *S. ventriculi* with metaplasia. Medical treatment with proton pump inhibitors, pro-kinetics, ciprofloxacin, and metronidazole failed to improve his symptoms. Finally, the patient was managed surgically with distal gastrectomy with Roux en Y reconstruction, and gastrostomy tube placement was done with satisfactory improvement in his symptoms.

## Introduction

*Sarcina ventriculi* is an anaerobic gram-positive coccus that can survive at extremely low pH levels. John Goodsir first described it in 1842. Since then, only a few cases have been reported [1]. *S. ventriculi* belongs to the family of Clostridiaceae. This pathogen gets its name from the Latin word “Sarcina,” which means “package.” It is usually built-in tetrads or octets [2]. Most patients with *S. ventriculi* present with gastrointestinal (GI) symptoms that vary from nausea, vomiting, and delayed gastric emptying to emphysematous gastritis, perforation, peritonitis, and gastric wall necrosis [3]. Endoscopy reveals retained food particles, gastric ulcers, and inflammation or erosions [4]. Here, we present a case of *S. ventriculi* with a long history of GI symptoms, including nausea, vomiting, early satiety, and delayed gastric emptying that required surgical intervention after the failure of medical treatment.

## Case presentation

**Figure 1:**
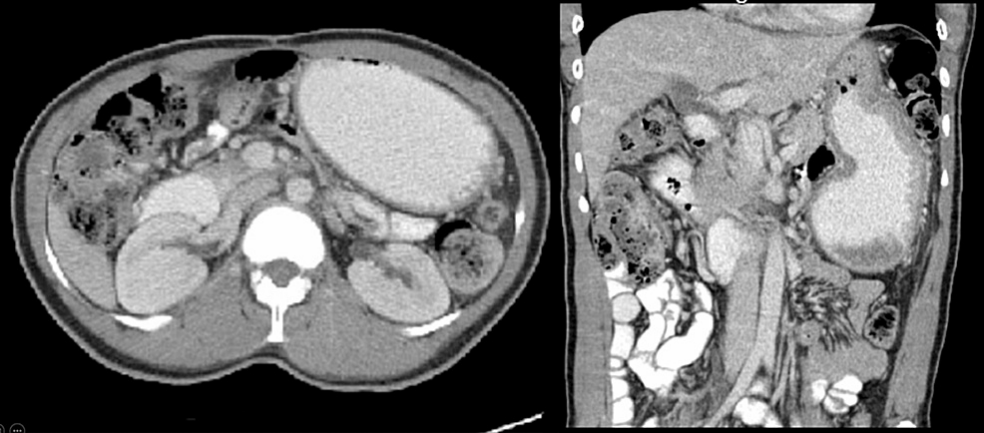
Abdominal CT showing dilated stomach with collapsed proximal duodenum suggesting gastric outlet obstruction.

**Figure 2:**
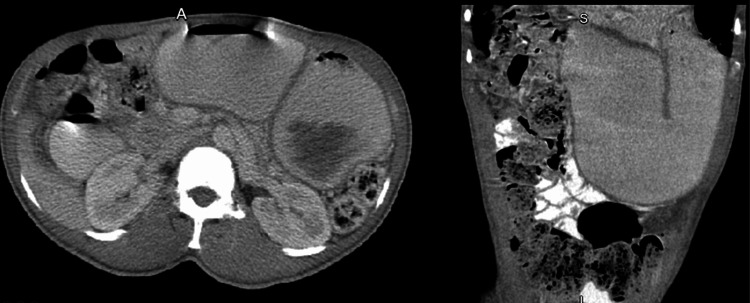
Abdominal CT revealing dilated stomach indicative of gastric outlet obstruction and/or gastroparesis.

**Figure 3:**
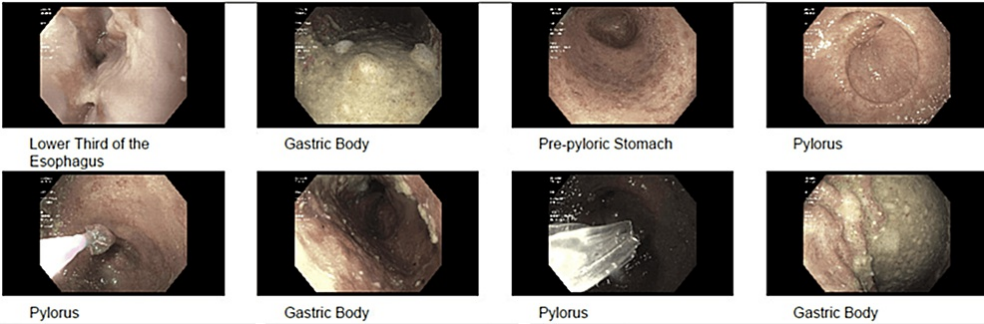
Esophagogastroduodenoscopy revealing grade B gastroesophageal reflux disease and a significant amount of residual food with fluid, along with pylorus stenosis

We present the case of a 43-year-old male patient from Nigeria with a history of schizophrenia. He presented to the emergency department (ED) with abdominal distension, nausea, repeated vomiting, and early satiety. He had waxing and waning symptoms for 10 years which worsened over a few months before presentation. On admission, he was not tolerating any food, and could only tolerate small amounts of juices that sometimes induced vomiting. He had lost more than 20 pounds over a couple of months. The patient had been hospitalized multiple times with similar symptoms with less severity. On examination, the patient was weak, awake, alert, and oriented to time, place, and person. Abdominal examination revealed a soft, swollen abdomen with diffuse tenderness in all quadrants, severe distension with hyperactive intestinal sounds, and no rigidity or hepatosplenomegaly. During the first admission five years ago, computed tomography (CT) of the abdomen with contrast revealed dilated stomach with collapsed proximal duodenum suggesting gastric outlet obstruction (Figure 1). Esophagogastroduodenoscopy (EGD) showed a small-sized hiatal hernia, multiple superficial ulcers at the body and antrum of the stomach with marked decreased contractility, dilated stomach full of fluid and food particles, and narrowed pyloric ring that prevented further passage of the scope without mass lesions. Multiple biopsies were taken and endorsed severe, active, chronic non-specific gastritis without metaplasia, negative for *Helicobacter pylori*. The patient was managed conservatively with prokinetics and proton pump inhibitors (PPIs). He showed satisfactory improvement in his symptoms. Four years later, the patient saw his gastroenterologist again for similar symptoms, with the main concern of appetite, fullness, and recurrent vomiting. A CT scan of the abdomen was performed at this time and revealed a dilated stomach indicative of gastric outlet obstruction and/or gastroparesis (Figure 2). An EGD was performed which revealed gastroesophageal reflux disease grade B, a significant amount of residual food with fluid, and pylorus stenosis where a biopsy was obtained (Figure 3). The patient was scheduled for another session of EGD for the possibility of dilatation. An EGD revealed a dilated, not contractile stomach with a large amount of food remnants. A pediatric colonoscopy was used to pass through the enormously dilated stomach using the through-the-scope (TTS) dilator and triamcinolone injection, revealing a normal duodenum once the stenosed area was traversed. The surgical biopsy culture revealed *S. ventriculi* associated with gastritis and metaplasia, negative for *H. pylori* and dysplasia. Antibiotic therapy with metronidazole, ciprofloxacin, high-dose PPI, and prokinetics was started.

**Figure 4:**
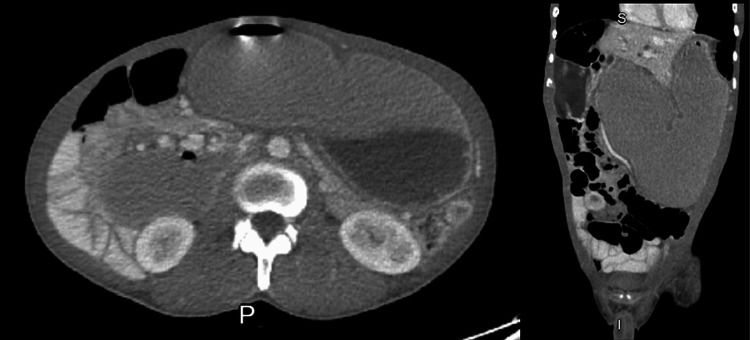
CT abdomen showing extensive abdominal dilatation.

The patient reported mild improvement and was referred for follow-up and scheduled for EGD sessions for dilatation. Eight months later, the patient presented at the ED again with the same symptoms. Lab tests revealed electrolyte imbalance, iron deficiency anemia, and mild elevations in lipase and transaminitis. A CT scan of the abdomen revealed extensive dilatation of the stomach (Figure 4). Surgery was consulted, and laparoscopic gastric bypass was recommended (gastro-jejunostomy). A distal gastrectomy was performed with Roux-en-Y reconstruction and placement of a gastrostomy tube. The diet was gradually advanced and tolerated. Surgical pathology revealed mixed bacteria, including *S. ventriculi* and other bacteria, budding fungi, and non-necrotizing granulomas in the gastric wall and 13 removed lymph nodes. Infectious diseases was consulted and recommended against antifungal treatment unless fever developed. The serum angiotensin-converting enzyme and vitamin D levels were within normal ranges. The chest CT was normal, and sarcoidosis was ruled out. Upon discharge from the hospital, the patient was in fair condition and was scheduled for follow-up appointments with the gastroenterology, infectious diseases, and surgery clinics.

## Discussion

The disease-causing role of *S. ventriculi* in humans is still uncertain and controversial [4]. Several studies have found that the organism is linked to delayed gastric emptying. Overgrowth is facilitated by concurrent underlying conditions that promote gastric retention, such as diabetic gastroparesis, pyloric stenosis, and gastric surgery [5]. Obtaining a biopsy for lymphadenopathy with *S. ventriculi* is important to exclude other causes of the condition, such as malignancy or sarcoidosis [6,7].

Nevertheless, even though the pathogenicity of *S. ventriculi* in humans remains unclear, some authors favor the theory that *S. ventriculi* causes its effect directly on a healthy stomach [8], This may be explained by the direct regional deposition of acetaldehyde and ethanol formed by the pathogen during fermentation of carbohydrates which might induce stomach and duodenal damage similar to acetaldehyde-induced mucosal damage in severe alcohol intake. Furthermore, many patients experience abdominal distention as a result of the carbon dioxide produced by glucose fermentation and pyruvate metabolism [9]. On the other hand, Canale et al. [10] mentioned that SV grows in the human gut as a result of delayed gastric emptying precipitated by diseases such as diabetic gastroparesis, gastric reconstructive surgery, scarring, and pyloric stenosis. Possibly, it induces its effect by a combination of pre-existing mucosal damage and delayed gastric emptying, which increases the accessibility of nutrient substrates. Furthermore, their fermentative carbohydrate metabolism, which produces acetaldehyde and ethanol, can clarify the emphysematous damage that frequently complicates this type of infection [11].

*S. ventriculi* has been reported in both pediatric and adult populations, although less reported in pediatrics but is much more likely to develop emphysematous gastritis and perforation in such populations [5]. The first case of emphysematous gastritis was reported by Tolentino et al. in 2003 [9] in a 14-year-old male patient who presented with abdominal pain and distension found to have gastric perforation and necrotic stomach in the fundus and greater curvature on laparotomy. Since 2003, few cases have been reported in the literature. In 2022, a systematic review by Tartaglia et al. [12] reported 65 cases from 55 articles with abdominal pain and gastric distension as the main presenting symptoms, with a median age of 51 years at presentation, diagnosed mainly by histopathological examination of endoscopic biopsies taken from the stomach. Esophageal *S. ventriculi* has been reported only in 15 cases in the literature, with food impaction being a major finding in most of these cases during endoscopy, which may be related to the effect of *S. ventriculi* on esophageal motility [13].

There is no consensus about a possible standard treatment regimen and its duration for *S. ventriculi* [12]. The main antibiotics used to treat *S. ventriculi* are metronidazole alone or metronidazole plus ciprofloxacin [4]. Other antibiotics have shown efficacy in eradication as vancomycin and piperacillin-tazobactam [14]. Medlicott et al. [15] recommended the use of PPI and prokinetics for symptomatic improvement of gastric distension and gastroparesis in positive *S. ventriculi* patients. As in our case, surgery has been reported as a last resort intervention in many cases with *S. ventriculi* with no improvement after symptomatic treatment and antibiotics [16-18].

## Conclusions

This case warns clinicians that abdominal pain, vomiting, and CT findings of the dilated stomach can indicate *S. ventriculi*, especially with long-standing symptoms not responding to symptomatic treatment. Additionally, we emphasize the role of surgery as an option for treatment in such cases.
